# Engineering 3D copper foam current collectors: modification strategies and challenges toward stable lithium metal batteries

**DOI:** 10.1080/14686996.2025.2525064

**Published:** 2025-06-30

**Authors:** Dong-Run Yang, Qingsong Lai, Yu-Tong Long, Xu Shi, Yue Lu, Zhao-Meng Liu, Xuan-Wen Gao, Wen-Bin Luo

**Affiliations:** Institute for Energy Electrochemistry and Urban Mines Metallurgy, School of Metallurgy, Northeastern University, Shenyang, Liaoning, China

**Keywords:** Copper foam, current collector, lithium-ion batteries, lithium dendrites, lithium metal

## Abstract

Lithium metal is a promising anode for high-energy batteries due to its high capacity and low density. However, issues like dendrite growth and volume expansion limit its practical use. To address these challenges, three-dimensional (3D) copper foam current collectors with porous architectures and superior electrochemical properties have emerged as a research focus. Three-dimensional copper foam current collectors have emerged as a strategic solution, leveraging their porous architecture to regulate lithium nucleation, enhance mechanical stability, and maintain electrochemical equilibrium. Despite their potential, current implementations confront four key constraints: excessively large pore sizes, uneven surface current distribution (leading to non-uniform lithium deposition, dendrite growth, and dead lithium formation), poor lithiophilicity, and weak oxidation resistance. These factors hinder the long-term suppression of lithium dendrites and degrade the oxidation resistance of copper nanostructures. This review systematically examines recent advancements in 3D copper foam engineering through three principal modification approaches: metallic/alloy coatings, surface functionalization, and structural optimization. The advantages, limitations, and critical issues of these approaches are analyzed. Furthermore, the importance of 3D copper foam current collectors in advancing lithium metal batteries is elucidated, highlighting current achievements, areas for improvement, and potential applications. Finally, recommendations and future prospects for further optimization of 3D copper foam current collectors are proposed to achieve commercially viable lithium metal batteries.

## IMPACT STATEMENT

This review assesses 3D Cu foam modifications-coatings, functionalization, structural optimization-to enhance Li dendrite suppression, lithiophilicity, and cycling stability for viable high-energy metal anodes.

## GRAPHICAL ABSTRACT

**Figure:**
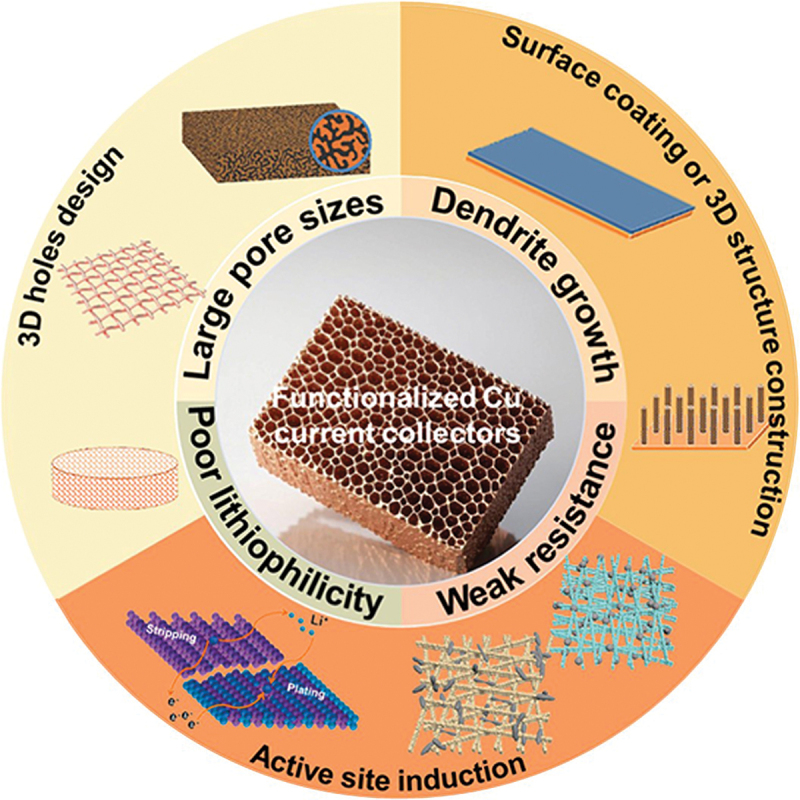

## 1. Introduction

**Figure 1.**
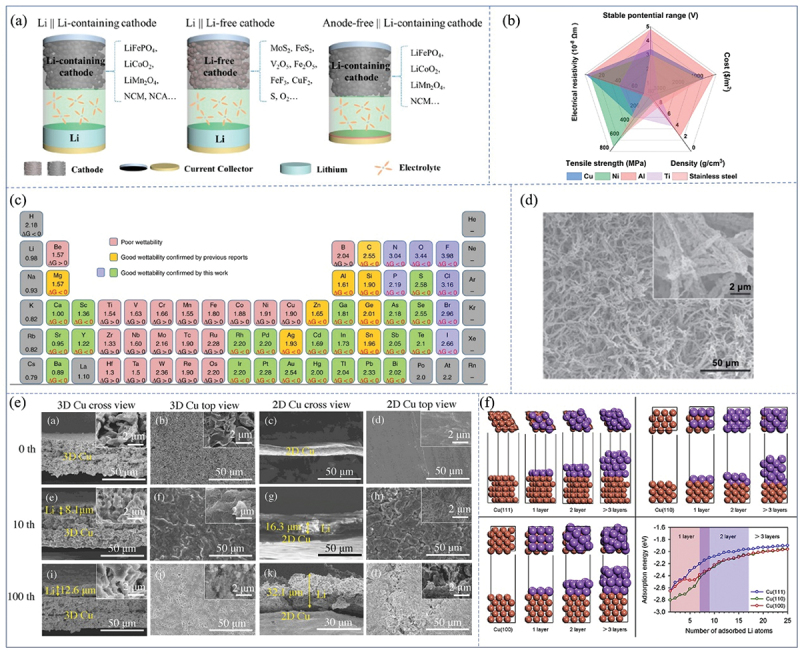
(a) Three common types of lithium metal batteries [12]. Reproduced by permission from [12], copyright [2021, American Chemical Society] (e) cross-view and top-view SEM images of 3D Cu foam and 2D Cu foil collectors before and after deposition of 2 mAh cm^–2^ Li metal at 1 mA cm^–2^ [12]. Reproduced by permission from [12], copyright (2021) American Chemical Society. (f) simulation of the Li deposition behaviors on the Cu surface and adsorption energy of the Li atoms on the Cu (111), (110) and (100) planes [36]. Reproduced by permission from [36], copyright (2019) Elsevier.

The pursuit of high-performance energy storage systems has intensified to power advanced electronics requiring extended battery life, driving significant interest in green, high-density energy solutions [1–3]. Among various rechargeable batteries, lithium-ion batteries have become ubiquitous in applications ranging from electric vehicles to portable devices, owing to their superior eco-friendliness, safety, and practicality [4–8]. These systems’ performance fundamentally depends on electrode, which dictates key metrics including energy density, power density, and cycle stability [9–11]. Lithium metal exhibits exceptional theoretical specific capacity (3860 mAh g^−1^) and ultralow density (0.59 g cm^−3^), making lithium metal batteries (LMBs) with lithium as the anode a focus for advancing energy density [12–14]. Current LMBs are primarily categorized into three types, as illustrated in Figure 1(a). The first two systems employ metallic lithium on the anode side and lithium-containing or lithium-free materials on the cathode side. However, in both systems, the Li^+^ inventory is significantly excessive, and surplus lithium metal reduces the overall energy density. In the third anode-free lithium metal battery, all lithium originates from lithium-containing cathode materials, enabling energy density exceeding 450 Wh kg^−1^. Yet, due to persistent side reactions, rapid capacity decay occurs as limited active lithium is consumed. Although LMBs hold strong theoretical promise, critical challenges in current systems continue to impede their practical deployment [15–18]. First, during charge-discharge cycles, the volumetric expansion of lithium metal induces structural instability in batteries. Second, the propensity for lithium dendrite formation during metal deposition risks internal structural damage and potential safety hazards. Third, limitations in the stability of solid electrolyte interphase (SEI) layers formed by lithium-electrolyte reactions hinder broader application of lithium metal batteries [19–22]. As a critical component of electrodes, current collectors not only support active materials but also collect electrons involved in electrochemical reactions, transport electrons between electrode materials and external circuits, and dissipate heat generated during electrochemical processes, thereby playing a decisive role in the release capability of electrode materials. Therefore, high-quality current collectors should exhibit high electronic conductivity, ionic insulation, electrochemical inertness, high mechanical strength, and low cost.

**Figure 2.**
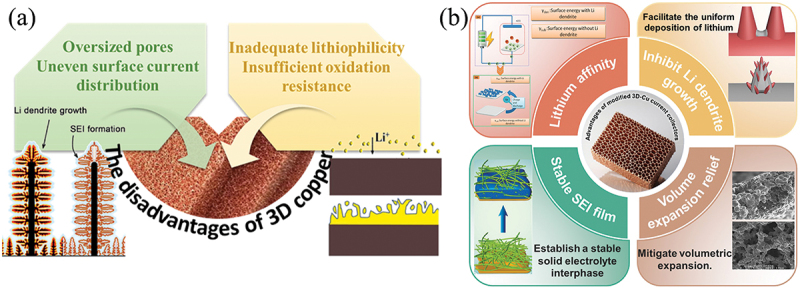
(a) The disadvantages of 3D copper; (b) advantages of modified 3D copper foam Collectors.66.

Currently, five primary metal-based materials have been proposed for current collectors: aluminum (Al), copper (Cu), nickel (Ni), titanium (Ti), and stainless steel. A comparative analysis of their key properties relevant to current collector performance is presented in Figure 1(b), summarized as follows: In terms of electrical resistivity, the order is Cu (1.7 × 10^−8^ Ω·m) < Al (2.82 × 10^−8^ Ω·m) < Ni (6.8 × 10^−8^ Ω·m) < Ti (42 × 10^−8^ Ω·m) < stainless steel (72 × 10^−8^ Ω·m). For tensile strength at room temperature (10–20 μm thick foils), the ranking is Al < Ti ≈ Cu < stainless steel < Ni. Regarding density, Al (2.7 g/cm^3^) < Ti (4.51 g/cm^3^) < stainless steel (7.9 g/cm^3^) < Ni (8.9 g/cm^3^) < Cu (8.96 g/cm^3^). In terms of cost, Al (130 USD/m^2^) < Cu (640 USD/m^2^) < Ni (795 USD/m^2^) < stainless steel (842 USD/m^2^) < Ti (3100 USD/m^2^). Finally, with respect to lithium affinity, only aluminum exhibits lithiophilicity, while all other materials display lithiophobic characteristics (Figure 1(c)) [23]. Based on these parameters, both aluminum and copper are considered suitable current collector materials. However, due to aluminum’s tendency to form alloys with lithium at low potentials, copper remains the most suitable option for metal battery anodes. Nevertheless, commercial planar copper foils exhibit lithiophobic properties, insufficient specific surface area, and non-uniform surface roughness. These factors collectively exacerbate the inhomogeneity of local current density and contribute to the aforementioned issues [24–29]. To address these challenges, initial efforts focused on replacing conventional copper foil with copper foam-specifically, highly porous copper featuring interconnected, foam-like pores-to accommodate the volumetric expansion of lithium metal. The scanning electron microscopy (SEM) image of copper foam in Figure 1(d) visually illustrates this porous architecture. Although post-cycling SEM analysis reveals that the 3D structure alleviates fluctuations in current density and volume expansion, the observed difference in cycling stability is not particularly significant, with improvements primarily in kinetics and capacity [30–35]. This may correlate with the nucleation and delithiation energy of lithium on the current collector. Subsequent theoretical studies demonstrate that among the (100), (111), and (110) facets commonly exhibited by copper, lithium preferentially deposits on the (110) and (100) facets (Figure 1(f)). However, since the (111) facet possesses the lowest surface energy, commercially available copper current collectors whether 2D or 3D are predominantly dominated by the (111) facet [36]. Consequently, although the design of three-dimensional current collector architectures to achieve uniform lithium deposition and suppress dendrite growth has emerged as a critical research frontier, persistent challenges continue to hinder the advancement of 3D copper foam – specifically referring to copper foams with substantial thickness and a more pronounced three-dimensional structure [37]. Moreover, excessively large pores and non-uniform surface current distributions exacerbate lithium deposition irregularities, promoting dendrite growth and the accumulation of dead lithium. Additionally, copper nanostructures – referring broadly to copper materials with nanoscale features – exhibit insufficient lithiophilicity and poor oxidation resistance, which further constrain their long-term effectiveness in suppressing dendrite growth (Figure 2(a)).

Systematic evaluation of modification strategies-encompassing metal/metal compound functionalization, surface treatments, and structural optimization-is therefore imperative (Figure 2(b)). This review systematically investigates recent advancements in 3D-Cu current collectors for lithium metal batteries, providing an in-depth analysis of three primary modification strategies alongside rigorous evaluations of their respective merits and limitations. Distinguished from prior studies, our work synergistically integrates material performance analysis with engineering design challenges, methodically exploring critical bottlenecks in 3D-Cu implementation for lithium metal batteries. By elucidating the mechanistic role of three-dimensional architectures in battery technology advancement and identifying crucial knowledge gaps, we propose targeted optimization strategies that stimulate the design of high-performance current collectors while enhancing their practical viability.

## 2. Copper foam current collector modification methods

### 2.1. Metal or metal compound modification

Due to the inherent structure of copper foam, it offers significant advantages over traditional copper foil, especially in achieving stable lithium deposition. To facilitate this process, it is essential to introduce lithium-affinity components that reduce the nucleation overpotential and deposition resistance on the surface of copper foam. Consequently, extensive research has focused on surface chemical or structural modifications of copper foam, particularly through metal or metal compound modifications. This approach involves coating or depositing metals or metal compounds, such as silver, zinc, and copper compounds, onto the copper foam current collector. The primary goal of this modification is to enhance the affinity between the current collector and lithium, thereby lowering the nucleation and deposition overpotentials. The application of such techniques has been shown to effectively suppress the formation of lithium dendrites and dead lithium, which are significant challenges in the operation of lithium-metal batteries. These issues adversely affect the performance and safety of the batteries. By applying metal or metal compound modifications, these challenges can be mitigated, thereby improving the performance of lithium-metal batteries. The 3D structure of copper foam, combined with alloying modifications, results in a smoother lithium deposition surface, with alloyed lithium exhibiting higher surface energy, which helps to slow down dendrite formation and ensure stable lithium deposition [38]. This provides a theoretical foundation for surface modifications of copper foam. As a result, modifying copper foam with metals or metal compounds that form alloys with lithium can convert the entire current collector into a lithium-friendly framework. Enhanced affinity reduces the nucleation barrier for lithium and promotes uniform deposition behavior, effectively preventing dendrite formation and the accumulation of dead lithium.

**Figure 3.**
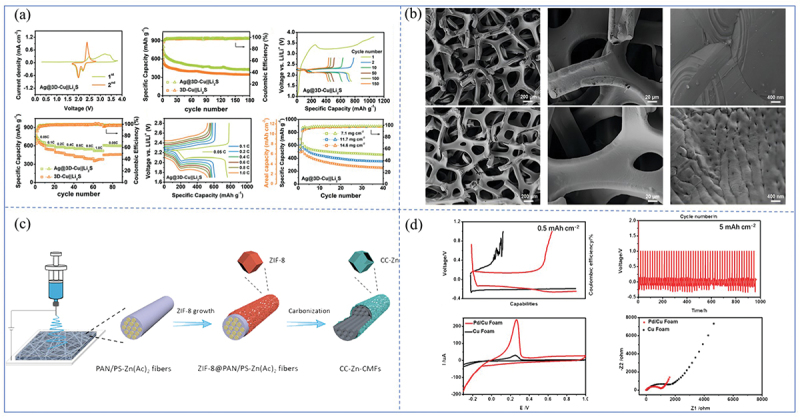
(a) Electrochemical performance tests of Ag@3D-Cu foams [39]. (b) SEM images of (b1-b3) 3D-Cu and (b4-b6) Ag@3D-Cu foams [39]. Reproduced by permission from [39], copyright (2021) American Chemical Society. (c) schematic diagram of the synthesis of CC-Zn-CMFs [42]. Reproduced by permission from [42], copyright (2021) Wiley. (d) electrochemical performance tests of each of the foam Pd/Cu and foam Cu [43]. Reproduced by permission from [43], copyright (2022) Elsevier.

Lithium-affinity silver (Ag) coatings represent an effective strategy for ensuring stable lithium deposition. Using thermal evaporation techniques, silver coatings of approximately 100 nm thickness can be applied to commercial copper foam substrates. As illustrated in Figure 3(a, b), the Ag@3D-Cu||Li_2_S anode-free battery exhibits stable cycling and rate performance, with an initial areal capacity reaching 7.4 mAh cm^−2^, even at a loading of 14.6 mg cm^−2^ [39]. Zhao et al. employed a displacement reaction under ultrasonic conditions to anchor silver nanoparticles onto the surface of copper foam, resulting in a lithium-affinitive Ag@CF electrode. This electrode not only reduces the nucleation overpotential (about 34 mV) for lithium atoms but also promotes uniform lithium deposition and enhances coulombic efficiency (no change after 350 cycles). It demonstrates ultra-long cycle life in both half-cell and symmetric cell configurations. Additionally, this design exhibited improved cycling performance in full batteries using LiFePO₄ and Li-Ag@CF electrodes. The displacement reaction under ultrasonic conditions aids in removing silver nanoparticles with weak adhesion, while ensuring that the remaining nanoparticles are tightly anchored to the CF surface. This guarantees the uniform and robust anchoring of silver nanoparticles on the copper foam substrate during prolonged cycles [40].

As a lithiophilic element, zinc coating serves as an effective modification strategy for copper foam, addressing the issue of non-directional and uneven lithium deposition caused by the foam’s irregular surface lattice structure. The electrospray templating method illustrated in Figure 3(c) enables precise deposition of zinc onto the copper foam through a removable template, thereby allowing for controlled uniformity and thickness of the coating [41]. Furthermore, other studies have reported the application of zinc metal films to the surface, facilitating the formation of a Li_x_Zn_y_ solid solution during lithium deposition, which buffers dendrite formation and suppresses their growth [42]. The zinc layer remains stable during lithium stripping, thus ensuring a prolonged and stable electrochemical cycling performance. In a relevant study, Wang et al. developed a dendrite-free, high-capacity anode for low-temperature potassium metal batteries by depositing a palladium (Pd) coating on copper foam. As illustrated in Figure 3(d), electrochemical performance tests of palladium/copper (Pd/Cu) foam and copper foam revealed that the Pd/Cu foam exhibits high potassium (K) affinity, which facilitates the regulation of potassium ion deposition behavior, thereby reducing the likelihood of dendrite formation. Pd/Cu foam facilitates the diffusion of potassium ions during charge and discharge processes, ensuring the proper redox reaction of the cathode material at low temperatures. Moreover, it reduces the reactivity between the potassium metal anode and the electrolyte, thereby suppressing side reactions and ensuring stable cycling [43]. However, further optimization of the electrode fabrication process is required to enhance the stability and cycling performance of the K/Pd/Cu composite anode. This Pd/Cu foam composite modification method holds promise for application in lithium-metal batteries, offering a novel approach for the use of copper foam current collectors.

**Figure 4.**
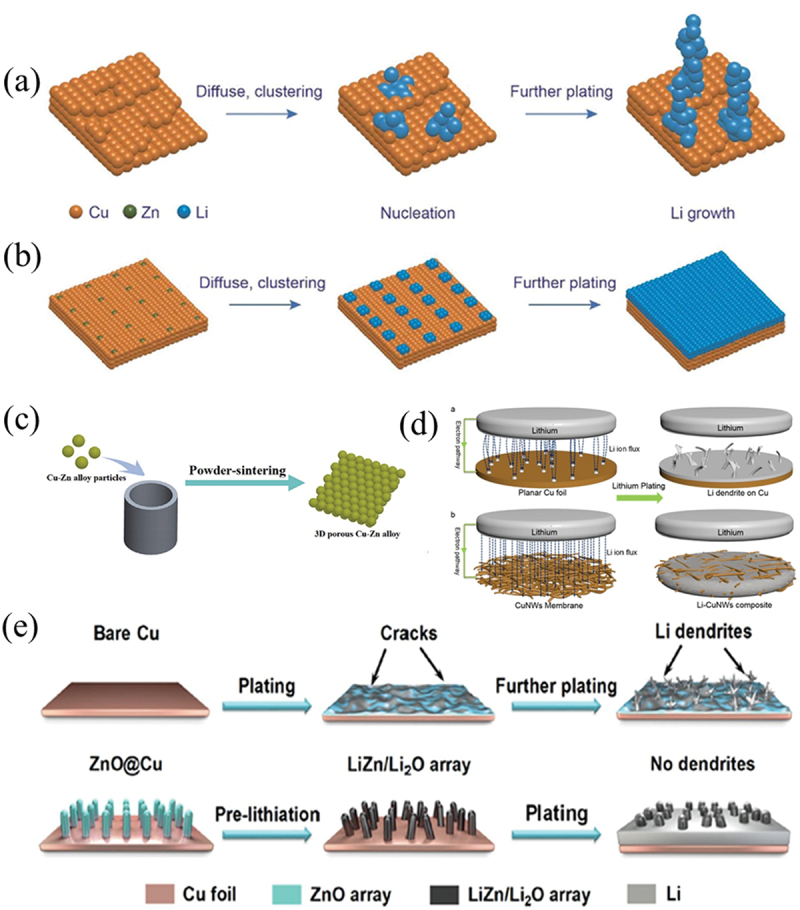
Schematic of Li plating process on (a) pure Cu, and (b) Cu99Zn substrates [46]. Reproduced by permission from [46], copyright (2018) Elsevier. (c) illustration of the powder-sintering process [47]. Reproduced by permission from [47], copyright (2019) Elsevier. (d) schematics of Li^+^ flux distribution and Li-metal plating models on different CCs. Planar Cu foil and Cu nanowire network. The distributions of Li^+^ flux is presented by dashed lines [50]. Reproduced by permission from [50], copyright (2016) American Chemical Society. (e) The gray parts on the CCs represent the plated Li. illustration of Li plating on the traditional Cu electrode, and lithiated ZnO@Cu electrode [49]. Reproduced by permission from [49], copyright (2019) Elsevier.

Ma et al. developed flower-like nickel oxide (NiO) grown on copper foam using hydrothermal and post-annealing methods. Lithium-affinitive NiO possesses a large surface area, providing abundant sites for lithium deposition and promoting a dendrite-free deposition process. Moreover, the combination of lithium-affinitive NiO with 3D copper foam alleviates the volume expansion of lithium, suppresses dead lithium formation, and enhances lithium metal utilization, leading to superior electrochemical performance [44]. Consequently, the NiO@CF composite exhibited a long stable lifespan of 1200 hours with a voltage hysteresis of 93 mV at a current density of 10 mA cm^−2^ in symmetric cells. Additionally, the full battery maintained 85% capacity retention after 500 cycles at 3 C. To reduce the lithium nucleation overpotential, alloying Cu with other metals can optimize the lithium deposition process. Yan et al. systematically investigated the lithium nucleation behavior on different metal substrates (e.g. Cu, Au, Zn) and proposed that lithium tends to deposit selectively on these specific metal substrates without nucleation barriers, particularly on metals exhibiting solubility in lithium [45]. Due to the observed substrate-dependent characteristics of lithium deposition, numerous strategies have been proposed to optimize the deposition process, among which Zn alloying of Cu current collectors (Cu CC) has garnered significant attention. This is primarily because Zn is a relatively low-cost and abundant metal. Additionally, Zn and Cu have similar atomic radii, enabling alloy formation at any atomic ratio without lattice mismatch, thereby ensuring uniform Zn distribution in the alloy [46]. Liu et al. modified Cu CC into a Cu99Zn alloy using magnetron sputtering technology (Figure 4(a, b)) [46]. The advantages of magnetron sputtering include precise control over the atomic ratio and thickness of the alloy film, as well as achieving superior surface smoothness. Fan et al. employed a powder-sintering method to fabricate a 3D porous Cu-Zn alloy (Figure 4(c)) [47]. The experimental procedure involved sintering Cu-Zn alloy particles at 600°C for 4 hours under an Ar/H₂ (95%/5%) atmosphere. The resulting 3D porous Cu-Zn alloy not only suppressed lithium dendrite growth by enhancing lithiophilicity but also increased the specific surface area by 36.6 times compared to 2D Cu foil, thereby reducing local current density. When paired with a Li foil counter electrode, the 3D porous Cu-Zn alloy electrode maintained a Coulombic efficiency (CE) of 98.3% over 160 cycles at 1.0 mA cm^− 2^, whereas the Cu foil electrode exhibited a CE below 80.0% after only 55 cycles. However, it should be noted that excessive specific surface area amplifies contact and reactions between lithium and the electrolyte, leading to increased lithium consumption during solid electrolyte interphase (SEI) layer formation.

Metal oxides can react with lithium to form Li₂O and metal-Li alloys. The metal-Li alloy enhances the lithiophilicity of the current collector (CC) by reducing the nucleation overpotential, while the generated Li₂O, a common inorganic component of the solid electrolyte interphase (SEI), homogenizes the electric field distribution and facilitates Li-ion migration [48,49]. Lu et al. developed a free-standing copper nanowire network CC decorated with ZnO nanoparticles to improve the compatibility between lithium and copper nanowires (Figure 4(d)) [50]. The incorporation of ZnO nanoparticles significantly lowered the lithium nucleation overpotential (~10 mV), while the nanowire structure guided a more uniform distribution of Li-ion flux. Wang et al. constructed a 3D Cu-ZnO CC featuring ZnO nanorod arrays on a planar Cu substrate (Figure 4(e)) [49]. Their study demonstrated that the CC not only provides uniform nucleation sites but also acts as a lithium host to buffer volume changes during cycling, thereby enabling homogeneous nucleation and reduced local current density.

### 2.2. Surface treatment modification

**Figure 5.**
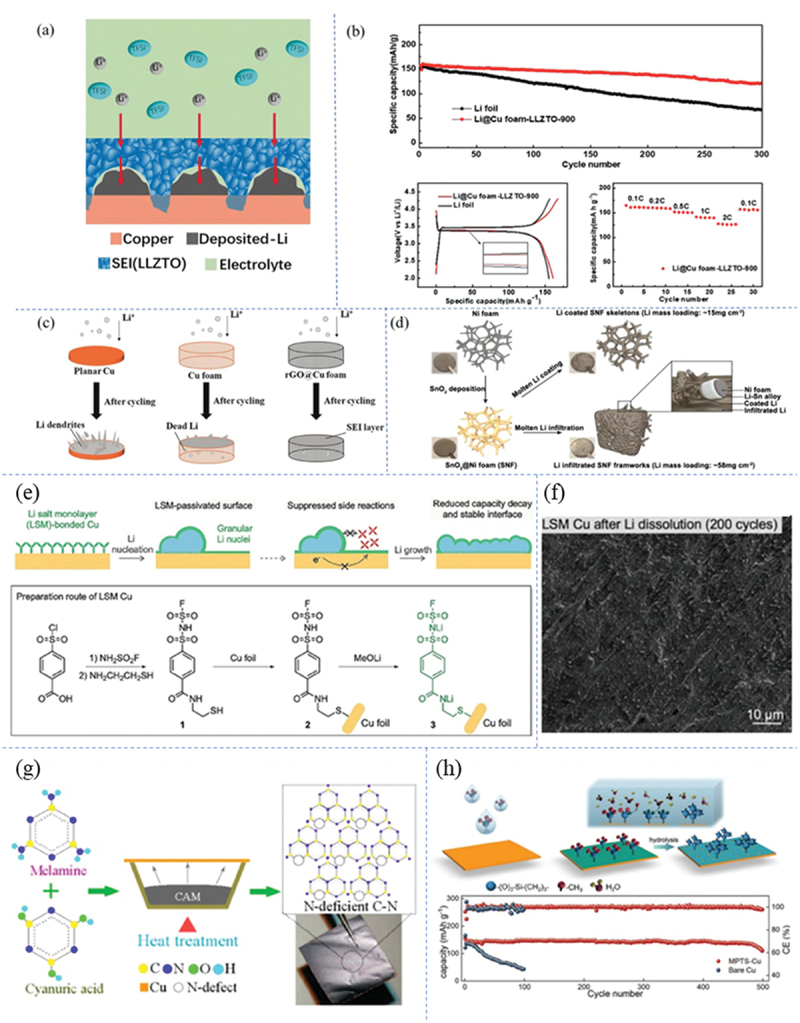
(a) Interfacial Li deposition and formation of homogeneous artificial SEI [51]. (b) electrochemical performance and charge/discharge curves of LiFePO_4_//Li@Cu foam-LLZTO-900 and LiFePO_4_//Li full cells [51]. Reproduced by permission from [51], copyright (2019) Wiley. (c) lithium deposition on bare planar Cu, Cu foam and rGo@cu foam [53]. (d) schematic of two different architectures for injecting molten Li into SnO_2_ to deposit Ni foam (SNF) hosts [24]. Reproduced by permission from [24], copyright (2019) Elsevier. (e) stable interface of a single layer of Li salt (LSM) bonded Cu for anode-free Li metal battery, and (f) SEM mages of Li dissolution after modification [54]. Reproduced by permission from [54], copyright (2023) Wiley. (g) growth process diagram of N-deficient C-N film on Cu foil [57]. Reproduced by permission from [57, copyright (2020) American Chemical Society. (h) schematic diagram of the preparation process of MPTS-Cu and the electrochemical performance of Li@MPTS-Cu||LFP and Li@bareCu||LFP battery at 0.5C [58]. Reproduced by permission from [58], copyright (2021) Wiley.

Surface modification of three-dimensional porous copper foam current collectors through the coating or immobilization of functional materials (such as graphene, artificial SEI layers, and metal nanoparticles) facilitates the formation of lithiophilic interfaces. The underlying mechanisms can be summarized in three key aspects (Figure 5(a)): (1) Functional coatings optimize charge transport pathways and regulate lithium-ion flux distribution, thereby promoting uniform lithium deposition; (2) Artificial SEI layers serve as physical barriers to prevent direct contact between the electrolyte and lithium metal, effectively suppressing side reactions and dendrite formation; (3) The three-dimensional porous architecture reduces the effective current density and provides ample space for lithium deposition, collectively mitigating the effects of volume expansion.

Building upon the aforementioned principles, researchers have developed a variety of innovative modification strategies. Li et al. employed a high-temperature sintering process to immobilize LLZTO solid-state electrolyte onto the surface of copper foam, resulting in a composite current collector with excellent interfacial stability and lithium-ion conductivity [51]. Compared with lithium foil, the Li@Cu foam-LLZTO-900 anode exhibits lower polarization voltage and higher capacity retention in full cells, and enables over 100 stable cycles when applied in lithium-oxygen batteries (Figure 5(b)) [51]. Yang’s group engineered a lithiophilic surface via graphene encapsulation, which not only reduces local current density but also significantly lowers lithium deposition overpotential due to its high electrical conductivity (about 40 mV). Experimental results demonstrated excellent cycling performance of the graphene-coated copper foam in both half-cell and symmetric cell configurations. When cycling at 0.5 mA cm^−2^, the current collector presents a CE as high as 98.6% for 250 cycles in half cells and a low voltage hysteresis of 10 mV over 2000 h in symmetric cells [52]. To further explore the regulatory mechanisms of three-dimensional structures on lithium deposition behavior, Yu et al. systematically compared the lithium deposition morphologies of planar copper foil, copper foam, and rGO@copper foam (Figure 5(c)) [53]. The rGO layer, formed through in situ reduction of graphene oxide prepared by the Hummers method, effectively suppresses dendrite growth and enhances battery cycle life. To mitigate irregular dendrite formation and the substantial volume changes associated with lithium metal anodes, Xia et al. developed two distinct lithiophilic architectures: a tin(IV) oxide (SnO_2_)-coated nickel foam (SNF) and a framework exhibiting superior wettability with molten lithium, as illustrated in Figure 5(d). Experimental findings reveal that both the Li-coated SNF skeleton and the Li-infused SNF framework substantially enhance cycling stability, improve electrode dimensional integrity, and accelerate lithium-ion charge transfer kinetics [24]. Notably, surface chemical modifications also demonstrate significant effects: Fu’s team applied a sodium formate solution post-treatment to obtain an antioxidant coating, which enabled the modified current collector to maintain excellent cycling performance with 74.8% capacity retention after 1000 cycles at 1 C [31]. Wu et al. achieved copper foil passivation via a monomolecular layer of lithium salts (Figures 5(e, f)), a strategy that not only reduces the energy consumption for SEI formation but also enhances the first-cycle efficiency (~20%) and long-term cycling life of soft-pack cells without a negative electrode [54]. In the field of interfacial engineering, researchers have accelerated the in situ formation of stable interfaces by incorporating key SEI components such as LiF [55] and Li_3_N [56]. For example, nitrogen-deficient C-N films prepared by thermal evaporation significantly enhance the conductivity of current collector functional layers (Figure 5(g)) [57]. Wen et al. innovatively constructed a flexible siloxane film via MPTS self-assembly (Figure 5(h)), whose unique chemical stability and lithiophilic properties ensure uniform lithium-ion flux, achieving nearly 500 stable cycles in full cells paired with high-capacity LiFePO_4_ cathodes [58].

In summary, surface treatment modification is an effective strategy for enhancing the electrochemical performance of 3D-Cu current collectors (i.e. copper-based current collectors with a three-dimensional structure), including cycling stability, coulombic efficiency, and battery energy density. This is primarily achieved through methods such as protective coating layers. Surface treatments optimize the surface properties of 3D current collectors, thereby improving lithium-ion transport efficiency and the performance of copper foam current collectors. This approach opens new possibilities for advancements in battery technology. However, several challenges remain in this field, including how to more effectively control the modification process and how to apply these modification strategies to a broader range of applications.

### 2.3. Structural modification

Surface structural modification is a crucial technological strategy that involves fabricating nanometer- or micrometer-scale structures with different morphology, size, porosity, and other structural parameters on copper foam current collectors, such as dendritic structures, fibrous structures, and so on. These carefully designed structures can effectively regulate the current density distribution, thereby providing accommodation space for lithium deposition, alleviating the volume expansion of the electrode, and enhancing the mechanical stability of the battery. Special surface structures can also increase the surface area, providing more nucleation sites for lithium. This means that lithium ions can form lithium metal at more locations, guiding uniform lithium deposition. Such uniform lithium deposition can prevent the formation of lithium dendrites, improving the safety and stability of the battery.

**Figure 6.**
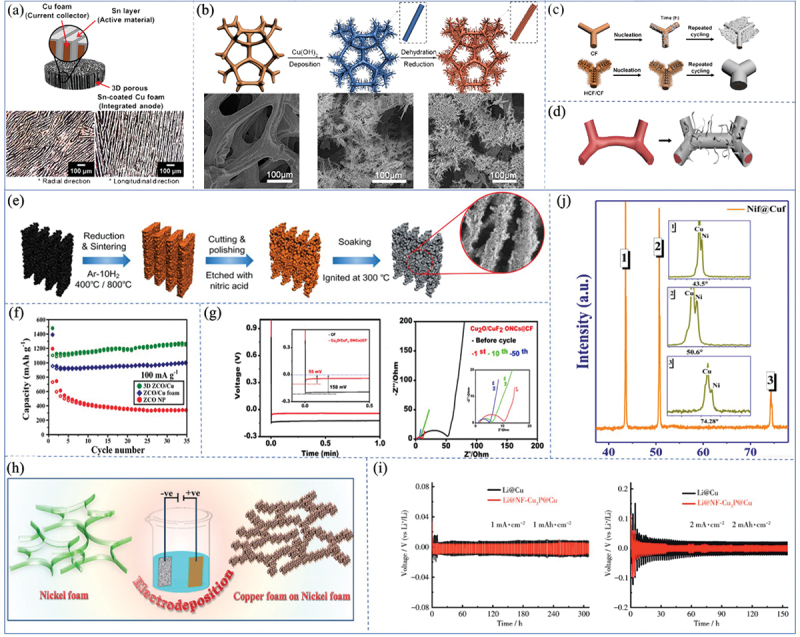
(a) Optical micrograph of 3D copper foam and schematic of 3D copper foam coated with the active material Sn by electroless plating [59]. Reproduced by permission from [59], copyright (2017) Elsevier. (b) schematic diagram of the HCF/CF current collector and corresponding SEM images [60]. (c) schematic of Li deposition on CF and HCF/CF collectors [60]. Reproduced by permission from [60], copyright (2020) Elsevier. (d) schematic of lithium dendrites grown on copper foam [61]. Reproduced by permission from [60], copyright (2024) Elsevier. (e) schematic of the preparation process of 3D ZCO/Cu electrode [62]. (f) cycling performance of 3D ZCO/Cu, ZCO/Cu foam and ZCONP at 100 mA g^–1^ [62]. Reproduced by permission from [60], copyright (2020) Springer Nature.(g) nucleation overpotentials of lithium-lithium deposition from Cu foam and Cu_2_O/CuF_2_ ONCs@Cu foam at constant current density of 1 mA cm^–2^ and Li@Cu foam in different cycles at a current density of 1 mA cm^–2^ ESI Nyquist measurement plots [63]. Reproduced by permission from [63], copyright (2023) American Chemical Society. (h) schematic of electrochemical growth of Cuf on nif [64]. (i) X-ray diffractograms of nif@Cuf [64]. Reproduced by permission from [64], copyright (2023) Elsevier. (j) Symmetric cells with Li@Cu and Li@Nf-Cu_3_P@Cu composite electrodes at different voltage cycling curves at different current densities and capacities:1 mA cm^–2^, 1 mAh cm^–2^, and 2 mA cm^–2^, 2 mAh cm^–2^ [65].

As shown in Figure 6(a), this is a schematic diagram of lithium deposition on copper foam and copper foam skeleton with secondary copper fiber [59]. The increased specific surface area helps suppress current density, enriches nucleation sites, promotes uniform lithium deposition, and suppresses dendrite formation. As shown in Figures 6(b, c), compared to conventional copper foam, Zhao et al. designed and successfully fabricated a 3D copper foam current collector featuring a surface copper fiber structure [60]. The HCF/CF skeleton demonstrates stable lithium plating/stripping behavior and high coulombic efficiency (98% retention rate after 200 cycles).The Li anode based on the HCF/CF current collector can operate for 820 hours at a current density of 1 mV cm^−2^ without significant voltage fluctuations [60].

Zhong et al. prepared nanometer-scale dendritic structures on the surface of copper foam current collectors, promoting more uniform lithium metal deposition and reducing dendrite growth, thereby extending the lifespan of the lithium metal anode [61]. The Cu dendritic structure facilitates the uniform deposition of lithium metal on the electrode surface. This is because the dendritic structure has a large surface area and excellent reactive sites, which favor uniform lithium deposition. The dendritic structure can significantly reduce the nucleation overpotential and deposition overpotential of lithium metal. This helps suppress the growth of lithium dendrites, thereby extending the lifespan of the lithium metal anode. Compared to commercial Cu foam, Cu dendrites with a layered structure exhibit lower lithium nucleation overpotential and deposition overpotential. This results in more stable cycling performance under constant voltage for Cu dendrite current collectors. Hyeji Park et al. prepared a novel 3D porous Sn-Cu structure as an anode material for advanced lithium-ion batteries. The micro-layered 3D porous foam with tin (Sn) as the active material was electroplated and used as the anode current collector. Compared to Sn-coated 2D copper foil, the 3D Sn-Cu foam with a zinc-coated micro-layer structure demonstrates excellent lithium-ion capacity and stable capacity retention. Additionally, this study also employed a freeze-casting process and a non-electroplating process to fabricate electrode materials, as shown in Figure 6(d). Mao et al. prepared a layered porous coral-like ZnO/ZnCo_2_O_4_/Co_3_O_4_ coating on a novel 3D through-plane copper current collector as shown in Figure 6(e). A layered copper current collector was synthesized via a one-step solution combustion method combined with directional freeze-casting, serving as a self-supporting anode for lithium-ion batteries (LIBs). During the combustion process, the rapid release of gases induces the formation of a coral-like, fluffy porous structure in the active material. The resulting three-dimensional ZCO/Cu anode exhibits a high reversible capacity and excellent cycling stability, as shown in Figure 6(f) [62]. Notably, the capacity gradually increases over repeated cycles-an unexpected phenomenon attributed to the following mechanism: (1) The pseudocapacitive behavior of a gel-like film, which forms reversibly between metal nanocrystals and amorphous Li_2_O, providing additional capacity during cycling for transition metal oxide (TMO) anodes. The presence of the porous coral-like structure, nanocrystals, and high-activity catalytic cobalt elements can trigger and further enhance this pseudocapacitive behavior. (2) During the charge-discharge cycle, the electrolyte infiltrates the nanopores of the porous coral-like active material, increasing the activation ratio of the electrode material. (3) With the cycling process, the synergistic effect between zinc-based oxides and cobalt-based oxides may become stronger, improving the conductivity of the electrode and accelerating the charge transfer kinetics.

Elumalai et al. developed a new 3D copper current collector. They proposed a two-step method to fabricate Cu_2_O/CuF_2_ octahedral nanocrystals (ONCs) onto a 3D Cu current collector. The resulting Cu foam with distributed ONCs provides active electrochemical sites, promoting uniform Li nucleation and dendrite-free Li deposition. The stable Cu_2_O/CuF_2_ ONCs@CF metal current collector is a reliable host for dendrite-free lithium metal anodes. In half-cell tests, lithium deposition at a constant current density of 1 mA cm^−2^ was observed. During the first lithium plating process, the voltage difference between the bottom-end drop and the following flat voltage platform (referred to as the Li nucleation overpotential) reflects the lithium affinity of the substrate [63]. The original CF exhibited a voltage drop of −158 mV at the initial nucleation overpotential, indicating a significant energy barrier during lithium deposition on its surface. This may be due to the poor affinity of lithium metal for the electroplating interface and the formation and growth of lithium dendrites. As shown in Figure 6(g), the Li@Cu_2_O/CuF_2_ ONC@CF electrode exhibited a smaller interfacial resistance after the initial cycle. Subsequently, after 50 cycles, the interfacial resistance decreased from 7.6 Ω (10 cycles) to 4.2 Ω. This significantly reduced the local effective current density, indicating that Cu_2_O/CuF_2_ ONCs have a larger electrochemical active surface area. For this reason, more stable electrode-electrolyte interface contact can be formed, which reduces charge transfer and internal resistance during the cycling process. Manisha Das et al. utilized the foam effect in foam structures, where copper foam electrochemically grows on foam nickel (Nif@Cuf) under controlled and optimized conditions, as shown in Figure 6(h). This bimetallic foam acts as a dual-function material, combining the roles of a conductive substrate and an active material. Extensive studies have been conducted on the electrochemical activity of the synthesized NiF@CuF to investigate and analyze its performance for water splitting [64]. It was found that the substrate exhibited good electrocatalytic properties, achieving a potential of 1.6 V with admirable stability, driving a current density of 10 mA cm^−2^. As shown in Figure 6(i), XRD analysis indicates the presence of metallic nickel and copper peaks with a cubic lattice structure, without the formation of any impurities or oxides. Wu et al. designed a Cu_3_P material with lithium affinity grown in situ inside 3D copper foam, which provides abundant nucleation sites for lithium, enabling rapid nucleation and uniform electroplating [67]. As shown in Figure 6(j), under various testing conditions, the voltage hysteresis of the Li@Nf‑Cu_3_P@Cu composite electrode in the symmetric cell is smaller than that of the Li@Cu composite electrode, indicating that the Li@Nf‑Cu_3_P@Cu composite electrode has excellent cycling stability.

**Figure 7.**
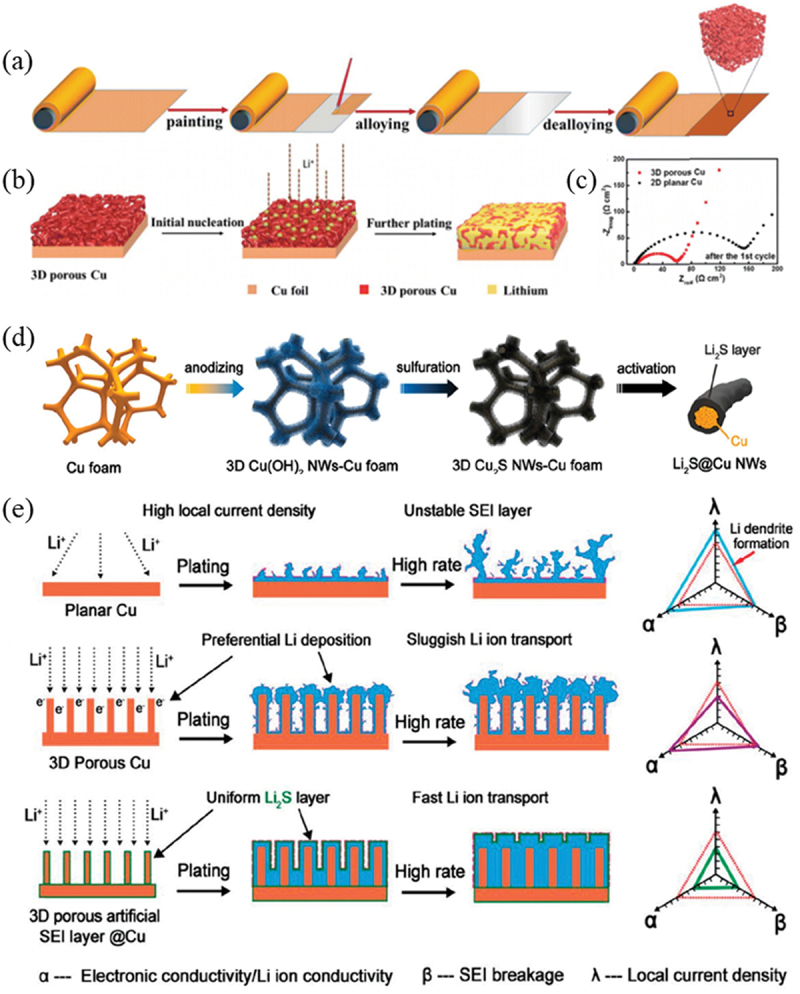
3D porous structured Cu foil. (a) Schematic illustration showing the preparation process of the 3D porous Cu from 2D planar Cu foil [66]. (b) schematic illustrations showing the structural changes in 3D porous Cu [66]. (c) EIS spectra of the 2D planar and 3D porous Cu CCs after 1 cycle [66]. Reproduced by permission from [66], copyright (2018) Royal Society of Chemistry. (d) schematic diagram of the preparation of Cu_2_S NWs-Cu foam.[68] (e) schematic diagrams of Li deposition behaviors on the different current collectors [68]. Reproduced by permission from [66], copyright (2020) Wiley.

Zhang et al. proposed a scalable fabrication route to construct a 3D porous architecture on commercial Cu foil via a liquid Ga-induced alloying-dealloying process (Figure 7(a)) [66]. The fabrication protocol involves: (1) uniformly coating liquid Ga onto the Cu foil surface, followed by annealing at 100°C to form a CuGa_2_ alloy phase; (2) selectively etching Ga to remove the alloying component, resulting in rearrangement of residual Cu atoms into a 3D nanoporous structure. Figure 7(b) illustrates the structural evolution of the 3D porous Cu during the initial nucleation and subsequent plating stages of lithiation. The porous skeleton, enriched with charge concentration centers, enables establishment of a uniform electric field distribution that facilitates current density homogenization and suppresses Li dendrite propagation. Figure 7(c) compares the interfacial impedance of different current collectors after initial Li plating/stripping cycles. The equivalent circuit modeling reveals that the 3D porous Cu-based half-cell exhibits significantly reduced combined resistance (R_SEI_ + R_ct_ = 54.9 Ω) compared to planar Cu foil (157.9 Ω), where R_SEI_ represents the solid-electrolyte interphase formation resistance and R_ct_ denotes charge transfer resistance. This optimized interfacial charge transport kinetics endows the 3D porous Cu with superior electrochemical performance in both symmetric cells and full-cell configuration.

Chen et al. employed a facile wet-chemical method to coat a 3D porous copper foam with a metal-organic framework (ZIF-8), which was subsequently modified via high-temperature treatment to obtain a nitrogen-doped carbon/zinc oxide composite-decorated copper foam current collector [67]. The 3D porous structure provides sufficient space for lithium metal deposition, where lithiophilic ZnO and nitrogen-doped carbon act as active sites to regulate uniform lithium metal deposition. As shown in Figure 7(d), Gong et al. constructed Cu₂S nanowire arrays (Cu₂S NWs-Cu) on copper foam through anodization and sulfidation treatments [68]. After initial lithiation, the nanowires exhibit increased average diameter due to in situ growth of Li_2_S layers. Figure 7(e) illustrates a comparative study of lithium metal deposition behaviors on different current collector surfaces [68]. For planar Cu foil, lithium deposition under high local current density readily induces lithium dendrite growth, while the 3D porous Cu structure effectively suppresses dendrite formation by reducing localized current density. However, the intrinsic lithiophobic nature of the Cu substrate leads to preferential lithium deposition at the top of the 3D porous Cu structure. In the case of the Li_2_S layer formed on the Cu_2_S nanowire-modified Cu current collector, it exhibits dual functions: (1) Promoting the formation of a stable solid electrolyte interphase (SEI); (2) Inhibiting preferential lithium deposition at the current collector surface or SEI layer defects by blocking continuous electrolyte-current collector contact, thereby preventing dendrite formation.

## 3. Conclusion and outlook

This paper summarizes the research methods of 3D copper foam current collectors in lithium metal batteries in recent years, including three main categories: metal or metal compound modification, surface treatment modification, and structural modification. It also analyzes the advantages and disadvantages of each modification method and the key issues existing in current research. The three modification methods aim to enhance the affinity between the copper foam current collector and lithium metal, suppress the growth of lithium dendrites, and improve the cycling stability of the battery. However, each method has its specific challenges and limitations. The summary of the corresponding performance is shown in Table 1.

**Table 1.** Summary of modified current collector materials and corresponding properties.

Modification material	Current density	Cycle Number	Ref
3D-Cu||Li_2_S	0.1 C	180	[39]
Li-Ag@CF	1 C	800	[40]
CC-Zn-CMFsLi//LFP	1 C	200	[42]
K/Pd/Cu foam	1 C	60	[43]
Li/NiO@CF‖LFP	3 C	500	[44]
Porous CuZn alloy	1 mA cm^−2^	160	[47]
Ni@Li2O-NW	1 mA cm^−2^	180	[48]
Lithiated ZnO@Cu	1 mA cm^−2^	200	[49]
Cu Nws	1 mA cm^−2^	200	[50]
ufoil-LLZT O-900	0.5 mA cm^−2^	600	[51]
GN@Cu foam	0.2 C	100	[52]
rGO @ Cu foam	1 mA cm^−2^	350	[53]
LSM Cu	3 mA cm^−2^	100	[54]
GBL-HC	1 mA cm^−2^	300	[55]
Li-CF@VN	1 mA cm^−2^	1000	[56]
g-C3N4	3 mA cm^−2^	450	[57]
Li@MPTS-Cu||LFP	0.5 C	480	[58]
Li@CF|LFP	2 C	500	[60]
3D ZCO/Cu	2 C	100	[62]
Ni-rich NMC|Li-Cu_2_O/CuF_2_ ONCs@Cu	0.1C	100	[63]
3D porous Cu	0.5 mA cm^−2^	200	[66]
MCuF	1 mA cm^−2^	600	[67]
3D Cu2S NWs – Cu foam	1 mA cm^−2^	500	[68]

Firstly, the material selection for metal or metal compound modification is limited. These materials need to meet the requirements of compatibility with lithium, conductivity, stability, and other factors, which brings certain difficulties in material selection. Additionally, such modifications may increase the weight and cost of the electrode, affecting both the performance and economic feasibility of the battery. Secondly, the surface treatment modification process may damage the original structure of the copper foam current collector, impacting its conductivity and porosity, which may negatively affect the battery’s performance. At the same time, the stability and compatibility of the materials may also be influenced, requiring strict control during the modification process. Finally, the preparation process of structural modification may introduce impurities or defects, potentially affecting its conductivity and stability. The optimization design of the structure must also consider factors such as cost, efficiency, and reliability, making the implementation of structural modification more complex.

**Figure 8.**
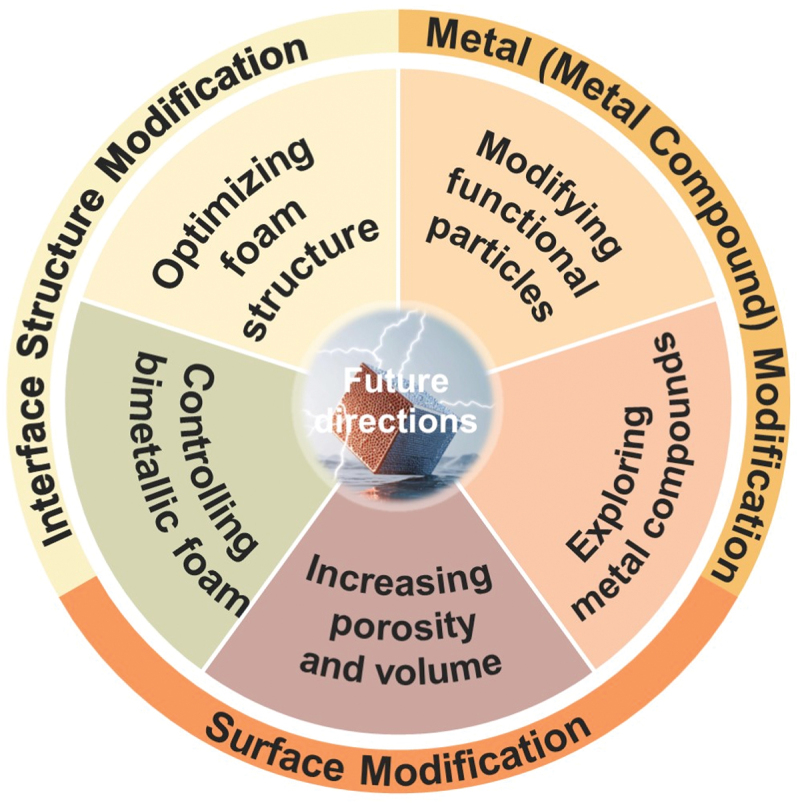
Prediction of future research directions and hotspots for 3D copper foam collectors.

In general, although each of these three modification methods has its own advantages, they also present certain challenges and limitations. Therefore, in practical applications, we need to comprehensively consider these factors based on specific needs and conditions and choose the most suitable modification method. Additionally, continuous research is necessary to overcome these challenges and further improve the performance and stability of the battery. This is a field full of challenges and opportunities, and we look forward to more researchers joining in to promote the development of this field. The outlook for the development of 3D copper foam current collectors is as follows Figure 8. Increasing Porosity and Pore Volume: To meet the demand for higher electroactive surface areas, the pore volume can be enhanced by increasing the thickness of the copper foam layer and designing more complex pore structures. By exploring different experimental methods for fabricating various copper foam materials, it is possible to obtain 3D copper foam structures with excellent electrochemical properties. This approach can effectively improve the performance of lithium metal batteries, enabling them to perform better in various applications. Optimizing Foam Structure: Investigating more advanced foam structures can further enhance the electrochemical performance of lithium metal batteries. By altering the structure of copper foam to increase its specific surface area and reduce local current density, the growth of lithium dendrites can be effectively suppressed, thus improving the reversible cycling efficiency and electrochemical performance of the battery. For example, electrochemical growth of copper foam on nickel foam (NiF@CuF) under controlled and optimized conditions, with the dual functions of the bimetallic foam, provides a new approach for optimizing the foam structure. Combining with Other Improvement Measures: Copper foam current collectors can work synergistically with other well-designed battery components, including lithium surface protection layers, electrolyte matching design, and the addition of multifunctional additives. By optimizing the electrolyte composition and additives, the cycling stability and reversible cycling efficiency of the battery can be enhanced. Furthermore, functional particles can be modified on the surface of copper foam to provide preferential nucleation sites, eliminate uneven electric fields, and suppress dendrite growth, thus improving battery performance. In addition to the aforementioned established design strategies, several emerging directions merit further exploration, such as bio-inspired architectures and AI-guided structural optimization. Specifically, the hierarchical porous structures found in natural organisms can be utilized to construct current collectors, while surface modifications mimicking honeycomb patterns or gecko foot hairs may enable controllable lithium adhesion to accommodate volume fluctuations. Moreover, machine learning algorithms can be employed to analyze the relationships between the geometric parameters of copper foam – such as pore size, porosity, and strut thickness – and the electrochemical performance of the battery. Based on predictive models, the optimal structure of copper foam can be designed to enhance lithium utilization and suppress dendrite formation.

In summary, copper foam current collectors are a critical component in lithium metal batteries and have a significant impact on battery performance. Many studies are currently focused on improving the performance of copper foam current collectors to enhance the Coulombic efficiency and cycling life of batteries. The application of copper foam current collectors in lithium metal batteries holds tremendous potential, and future improvements and developments will further drive the advancement of lithium metal batteries toward next-generation energy storage devices. Similarly, the application of 3D copper foam current collectors in other battery systems remains a subject for further research by scientists.
